# Analysis of circulating hem-endothelial marker RNA levels in preterm infants

**DOI:** 10.1186/1471-2431-9-42

**Published:** 2009-06-25

**Authors:** Tzipora Strauss, Sally Metsuyanim, Itai Pessach, Irit Shuchan-Eisen, Jacob Kuint, Benjamin Dekel

**Affiliations:** 1 Neonatal Department, Chaim Sheba Medical Center, Tel Aviv University, Tel Aviv, Israel; 2 Pediatric Stem Cell Research Institute Chaim Sheba Medical Center, Tel Aviv University, Tel Aviv, Israel; 3 Division of Immunology, Children's Hospital, Harvard Medical School, Boston, USA; 4 Department of Pediatrics, Chaim Sheba Medical Center, Sackler School of Medicine, Tel Aviv University, Tel Aviv, Israel

## Abstract

**Background:**

Circulating endothelial cells may serve as novel markers of angiogenesis. These include a subset of hem-endothelial progenitor cells that play a vital role in vascular growth and repair. The presence and clinical implications of circulating RNA levels as an expression for hematopoietic and endothelial-specific markers have not been previously evaluated in preterm infants. This study aims to determine circulating RNA levels of hem-endothelial marker genes in peripheral blood of preterm infants and begin to correlate these findings with prenatal complications.

**Methods:**

Peripheral blood samples from seventeen preterm neonates were analyzed at three consecutive post-delivery time points (day 3–5, 10–15 and 30). Using quantitative reverse transcription-polymerase chain reaction we studied the expression patterns of previously established hem-endothelial-specific progenitor-associated genes (*AC133, Tie-2, Flk-1 (VEGFR2) and Scl/Tal1*) in association with characteristics of prematurity and preterm morbidity.

**Results:**

Circulating *Tie-2 *and *SCL/Tal1 *RNA levels displayed an inverse correlation to gestational age (GA). We observed significantly elevated *Tie-2 *levels in preterm infants born to mothers with amnionitis, and in infants with sustained brain echogenicity on brain sonography. Other markers showed similar expression patterns yet we could not demonstrate statistically significant correlations.

**Conclusion:**

These preliminary findings suggest that circulating RNA levels especially *Tie2 *and *SCL *decline with maturation and might relate to some preterm complication. Further prospective follow up of larger cohorts are required to establish this association.

## Background

Survival of preterm infants has improved in the last decades thanks to advances in ventilation strategies, preterm nutrition and behavioral adaptation. Mortality and morbidity rates, however, are still high, especially in extremely low birth weight infants (ELBW) [1]. The three most common morbidities in preterm infants are: bronchopulmonary dysplasia (BPD), which is more prominent in infants born before 28 weeks of gestation and weighing less than 750–1000 g; retinopathy of prematurity (ROP), which is still the second most common cause of blindness among children, and germinal matrix intraventricular hemorrhage (GM-IVH) or periventricular leukomalacia (PVL) that may cause severe complications with long-term consequences, including post-hemorrhagic hydrocephalus, seizures, cerebral palsy and other neurological deficits. Vascular pathology is associated with all three conditions [2,3].

Blood vessel formation occurs through angiogenesis or vasculogenesis. Until recently, the term vasculogenesis described the process of blood vessel formation in the embryo. This occurs via differentiation of precursor cells (angioblasts) into endothelial cells, which then assemble into a primitive vascular network [4,5]. First isolated from peripheral blood in 1997 by Asahara *et al *[6], endothelial progenitor cells (EPCs), represent a heterogeneous population that expresses hematopoietic and endothelial-specific markers, including AC133, TIE-2, FLK-1 (VEGFR2) and SCL/TAL1.

Evidence that EPCs may be involved in endothelial maintenance and in neovascularization has led to the examination of the interaction between pathologic vasculopathy and EPC [7-9]. These studies proposed that the number of circulating EPCs is a surrogate biological marker for vascular function and cumulative cardiovascular risk [10,11] as well as predictive for outcome in ischemic stroke [12-15].

Only few studies involve EPCs in regard to premature infants and associated morbidities. Two studies suggested that modulation of EPCs may have therapeutic potential in BPD and ROP [16-18].

It was recently demonstrated that the quantification of circulating cells using quantitative reverse transcription PCR (qRT-PCR) of endothelial molecular markers is consistent with cell numbers obtained by flow cytometric analysis [19,20]. This technique provides an efficient tool for the detection and quantification of these cells similar to the widely used approach for the detection of circulating tumor cells in the peripheral blood [21-25].

To date no study has evaluated circulating RNA levels of hem-endothelial markers in preterm infants. In our present study we established a quantitative reverse transcription-polymerase chain reaction (qRT-PCR) for the evaluation of endothelial-specific progenitor-associated molecular markers e.g. (*AC133, Tie-2, Flk-1 (VEGFR2) and Scl/Tal1*) in preterm infants with different characteristics and in various time points in order to initially determine C-RNA levels and then provide data of possible association to prenatal complication.

## Methods

### Subjects

All preterm infants sequentially admitted to the neonatal intensive care unit between June 2006 and January 2007 with a gestational age (GA) less than 34 weeks were eligible to participate in the study. Infants with major congenital malformations were excluded. The study patients' characteristics are summarized in Table 1. Informed written consent was obtained from the parents. The study was approved by the ethical committee of the Sheba-Medical-Center.

**Table 1:** Patients characteristics

**GA**	**BW**	**Sex**	**Delivery**	**PNS**	**Mat amnionitis**	**MV**	**Surf**	**Sepsis**	**A' 1**	**A' 5**	**RDS**	**BPD**	**ROP**	**US**	**PVL**	**IVH**
26.6	888	M	PS	Y	N	Y	Y	N	2	9	Y	N	N	Y	N	N

26.4	880	F	CS	Y	N	Y	Y	N	4	8	Y	N	N	Y	N	N

25.4	724	M	CS	Y	N	Y	Y	Y	7	8	Y	N	Y	Y	N	N

27.4	1105	M	CS	Y	Y	Y	Y	Y	2	2	Y	N	N	N	N	N

29.4	980	F	CS	Y	N	Y	Y	N	5	10	Y	Y	N	Y	N	N

30.5	1325	F	CS	Y	N	N	N	N	9	10	N	N	N	N	N	N

29.4	1272	F	CS	Y	N	Y	Y	N	5	9	Y	N	N	Y	N	N

28.2	1115	M	PS	Y	N	Y	Y	Y	6	10	Y	N	N	Y	Y	Y

30.5	1550	M	CS	Y	N	N	N	N	9	10	N	N	N	N	N	N

31.6	1835	F	CS	Y	N	Y	Y	N	5	8	Y	N	N	N	N	N

33.2	1823	M	PS	Y	N	N	N	N	9	10	N	N	N	N	N	N

32	1378	M	CS	Y	N	N	N	N	6	8	N	N	N	N	N	N

31.6	1936	M	CS	Y	Y	N	N	N	6	8	N	N	N	N	N	N

32	1900	M	CS	Y	N	N	N	N	6	7	Y	N	N	N	N	N

31	1525	M	CS	Y	Y	N	N	N	8	9	N	N	N	N	N	N

32	2015	M	CS	Y	N	Y	Y	N	9	10	Y	N	N	N	N	Y

32	2160	F	PS	Y	N	N	N	N	9	10	N	N	N	N	N	N

**GA**-gestational age; **BW**-birth weight; **PNS**-prenatal amnionitis; **Mat amnionitis**-maternal amnionitis; **MV**-mechanical ventilation; **SURF**-surfactant; **A'1**-apgar score at 1 min; **A'5**-apgar sore at 5 min; **RDS**-respiratory distress syndrome; **BPD**-bronchopulmonary dysplasia; **ROP**-retinopathy of prematurity; **US**-head ultrasound **Y**-increased echogenicity **N**-normal US; **PVL**-periventricular leukomalacia; **IVH**-intraventricular hemorrhage; **M**-male; **F**-female; **Y**-yes; **N**-no; **PS**-partum spontaneous; **CS**-cesarean section.

### Study Protocol

1–1.5 milliliters of blood were drawn into a vacutainer containing 5.4 milligram potassium ethylenediaminetetra-acetic acid (kEDTA) on three consecutive time points after birth: day 3–5 of life, day 10–15 of life and at 1 month of life. The blood was immediately transferred to the laboratory. Expression of hem-endothelial-marker genes was measured by qRT-PCR (namely, *AC133, Tie-2, Flk-1 (VEGFR2) and Scl/Tal1*) as well as the angiogenic growth factor, VEGF.

### RNA isolation and cDNA preparation

Blood samples were lysed by Puregene RBC Lysis Solution (Gentra systems) to remove red cells, and total RNA was isolated using Trizol reagent (Invitrogen). cDNA was synthesized from total RNA using the Super Script First- Strand Synthesis System for PCR-RT kit (Invitrogen).

### Quantitative real-time reverse transcription-polymerase chain reaction

QRT-PCR was performed using a SYBR Green PCR Master Mix (Applied Biosystems). The qRT-PCR primers used is shown in Table 2. To eliminate DNA amplification during qRT-PCR we programmed primers that contained axon (splice) junctions. In addition, no template cell control (NTC) was checked in the real-time PCR reactions so as to ensure the lack of an amplification product indicative of DNA contamination of the reactions or the formation of primer dimer. Each PCR reaction contained 100 ng cDNA; Primer's final concentration was 500 nM each (forward and reverse). Amplification and detection were performed with the ABI7900HT sequence detection system (Applied Biosystem) and analyzed by the SDS 2.1 software. The thermal cycle used was 2 min at 50°C, 10 min at 95°C, and 40 cycles of 15 sec denaturation at 95°C with 1 min annealing and extension at 60°C. In order to evaluate target genes expression, Reverse quantitative (RQ) values were calculated for each sample. Briefly, the ΔCt value of the target gene was calculated by subtracting the Ct value of the reference gene (β-actin)-from the Ct value of the gene and then normalized against the results obtained from cord blood of full term infants (ΔΔCt method). All measurements were preformed in triplicate and an average result is presented

**Table 2:** PCR primers used in the present study

**Gene**	**Origin**		**Sequence**
**AC133**	Human	**F**	5'-TGGATGCAGAACTTGACAACGT-3'

		**R**	'-ATACCTGCTACGACAGTCGTGGT-3'5

**VEGF**	Human	**F**	'-AGCCTTGCCTTGCTGCTCTA-3'5

		**R**	'-GTGCTGGCCTTGGTGAGG-3'5

**VEGFR2 (FLK1)**	Human	**F**	'-GCATCTCATCTGTTACAGC-3'5

		**R**	'-CTTCATCAATCTTTACCCC-3'5

**SCL**	Human	**F**	'-AGCCGGATGCCTTCCCTAT-3'5

		**R**	'-CCGCACAACTTTGGTGTGG-3'5

**TIE2**	Human	**F**	'GCTTGCTCCTTTCTGGAACTGT-3'5

		**R**	'-CGCCACCCAGAGGCAAT-3'5

### Statistic

ANOVA analysis with repeated measurements was used to assess statistically differences in time course of gene expression levels. The average RQ of the patient groups were performed using analysis of variance (with confounders including GA, birth weight (BW), BPD, ROP, IVH, and PVL), and the Mann-Whitney test for comparison of parametric variables. P value for comparison between the groups was reported. Values of *p *≤ 0.05 was considered significant. All analyses were preformed using SPSS version 15 software (SPSS Inc. Chicago. IL, USA).

## Results

Twenty one preterm and eight full term infants entered our study. Three infants were excluded due to technical difficulties in RNA isolation and one infant died after 48 hours; therefore 17 preterm were eligible for the study. Table 1 shows the clinical and prenatal characteristics of the research group. Of the 17 preterm infants, 11 were males and 6 females with gestational age ranging between 25.4 and 33.2 weeks (average 29.9 ± 2.36 weeks) and BW between 724 and 2160 g (all were appropriate for GA, average 1436 ± 450 g). Thirteen of the infants were born by cesarean section delivery and four were partum spontaneous. None of the infants had any major congenital malformations. All mothers of the preterm infants had antenatal steroid therapy for lung maturation. One mother had proven amnionitis with positive cultures for enterococci in the placenta and another two mothers had suspected amnionitis (maternal fever and leukocytosis). Three infants had sepsis. Ten infants had respiratory distress syndrome (RDS) and nine of them were mechanically ventilated and treated with surfactant. Two infants were diagnosed with mild BPD and one with severe BPD. Two infants had patent ductus arteriosus (PDA) and were treated with indomethacin. One infant had ROP grade 3 and was treated with laser photocoagulation. Of the group of eight full term infants, 7 were born spontaneously and one by cesarean section due to breech presentation. All had normal pregnancy and follow up with intact hospitalization.

### C-RNA levels GA and BW

**Figure 1:**
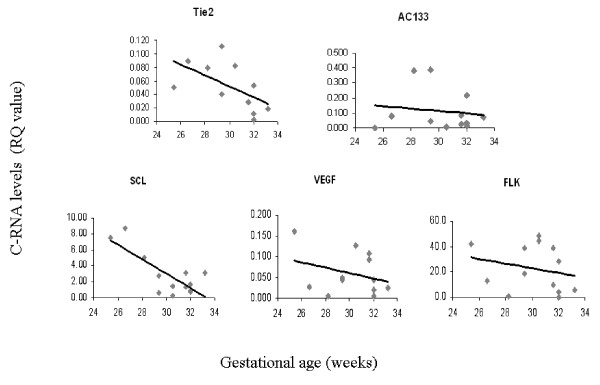
**C-RNA levels and gestational age**. Angiogenic marker levels were measured by qRT-PCR in preterm infants with a gestational age ranging from 24–34 wk as described under "Materials and Methods". Pearson correlation was used to assess the correlation of different angiogenic markers and gestational age. 5 distinctive markers were examined: Tie2 (A), AC133 (B), SCL (C), VEGF (D), and FLK (E). The figure depicts the measured RQ for each of the markers measured at 3–5 days after birth in comparison to GA (weeks).

**Figure 2:**
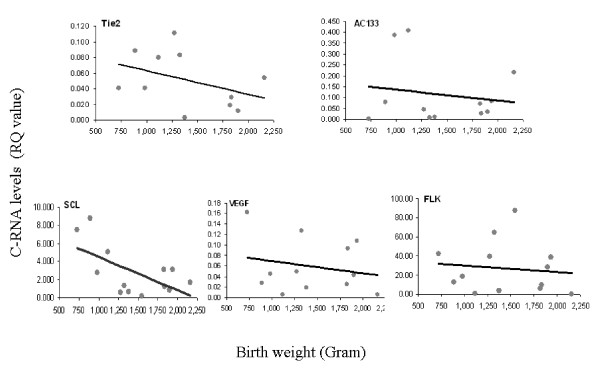
**C-RNA levels and birth weight**. Angiogenic marker levels were measured by qRT-PCR in preterm infants with a birth weights ranging from 724–2160 grams described under "Materials and Methods". Pearson correlation was used to assess the correlation of different angiogenic markers and BW. 5 distinctive markers were examined: Tie2, AC133, SCL,, VEGF, FLK. The figure depicts the measured RQ for each of the markers measured at 3–5 days after birth in comparison to BW (grams).

As GA and BW are important prognostic factors for preterm morbidity we looked for an association between C-RNA levels and these parameters. Only 10 infants had full results due to laboratory difficulties and insufficient amount of blood. There was a statistically significant inverse correlation between GA and circulating Tie2 (Figure 1, *r *= -0.597 *p *= 0.04 Pearson correlation) and SCL (Figure 1, *r *= -0.612 *p *= 0.026 Pearson correlation) mRNA levels. We found a tendency for an inverse correlation between BW and the expression levels of all C-RNA at 3–5 d after birth (Figure 2).

### C-RNA levels and brain echogenicity

**Figure 3:**
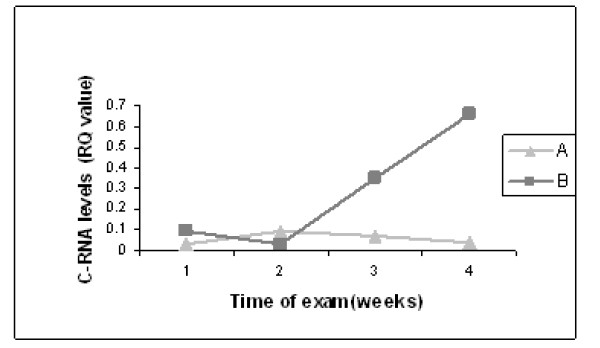
**Tie2 mRNA levels and increased brain echogenicity**. Serial head US scans were performed on all patients in the first week of life followed by a second one 10 days later and then a third at one month of age. Circulating Tie2 mRNA levels were measured at 3 time points after birth by qRT-PCR as described under 'Materials and Methods" in preterm infants with normal brain US (A) and in preterm infants with increased echogenicity on brain sonography (B). The data was analyzed using ANOVA analysis with repeated measurements.

Serial head ultrasound (US) scans were performed during the first month of life according to the protocol applied to all preterm infants admitted to our NICU: an initial ultrasound in the first week of life, followed by a second one 10 days later and then a third at one month of age. All three US were preformed and analyzed by the same pediatric radiology team. Six infants diagnosed with increased brain echogenicity on their first US: In four infants the increased echogenicity persist until discharge, one infant had additionally IVH grade I-II and in one infant the echogenicity resolved on his last exam. A time dependent elevation of Tie2 mRNA levels was observed in the six preterm infants diagnosed with brain echogenicity (Figure 3). The difference in Tie2 levels reached statistical significance at 1 month of age as compared to the levels of Tie2 expression in those infants with normal brain sonography, whose Tie2 expression levels remained relatively constant throughout the study period (0.66 ± 0.1 compared to 0.1 ± 0.15 p = 0.035)

### C-RNA levels and amnionitis

Three mothers were defined as suffering from clinical amnionitis (defined as maternal fever and leukocytosis during delivery) of which one had a positive placenta culture for enterococci. Although small number of patients- there was a significant elevation of Tie2 and AC133 expression levels in the blood of preterm infants born to mothers with amnionitis as compared to preterm infants born to healthy mothers as follows; Tie2: median of 0.05(0.03–0.2) in the absence of amnionitis compared to 0.5 (0.4–2.2) for mothers with amnionitis, *p *= 0.004 and AC133: median of 0.04 (0.002–0.4) in the absence of amnionitis compared to 0.9 (0.09–1.7) for mothers with amnionitis, *p *= 0.03)

### C-RNA levels in RDS and other preterm morbidities

Ten infants had evidence of RDS: nine were mechanically ventilated and treated with surfactant and the tenth was treated with 3 days of oxygen enrichment. No correlation was found between C-RNA levels and the presence of RDS, as could be expected due to the vascular independent pathophysiology that is attributed to RDS.

## Discussion

There is a fine balance between the extent of injury and the capacity of repairing endothelial damage and generating endothelial cells. Different environmental and inherited factors play a role in this process. Several studies in adults have shown that EPC levels may be a surrogate biological marker for angiogenesis and vasculogenesis as well as regeneration for vascular healing [7,8]. It was previously demonstrated that derivation of vessels in the post-natal kidney are from bone marrow-derived circulating cells [24,26]. Endothelial marker such as Tie2 has also been established to contribute to post-natal blood vessel formation [27,28]. Pieh *et al *have recently shown elevated plasma levels of the soluble receptor VEGFR-2 and soluble Tie2 in premature infants with active ROP [18]. In the present study we established qRT-PCR for the evaluation of hem-endothelial- associated molecular markers [21-23,26] in preterm infants. Of course, the mRNA expression level is not the same as the cell number of EPCs as few cells may express large amount of a given vascular marker. Nevertheless, our approach is based on a growing number of publications which have measured circulating RNA levels of hem-endothelial markers in vascular pathologies and cancer patients and have shown their levels to be consistent with FACS data enumerating circulating endothelial cells and EPCs [19-22]. Furthermore, Benjamin and colleagues have specifically addressed circulating tie-2 RNA levels measured by real-time PCR and were able to show it as a surrogate marker for vasculogenesis [23,29]. Thus, molecular analysis of circulating marker levels has the potential to reflect angiogenic potential at the tissue level. Quantitative molecular evaluation offers some distinct advantages. The most important of which is, that the quantification of RNA can be easily performed in large series of frozen samples and on small quantities of blood. These features are of special significance when studying preterm infants where blood samples are difficult to obtain and the amount retrieved is minimal. In addition, this method allows for better and more easily achieved inter-laboratory standardization.

This is the first report demonstrating that hem-endothelial-associated molecular marker levels may correlate with clinical parameters such as gestational age and birth weight. Notably, our examination of a wide GA range (25.4–33.2 weeks) demonstrated an inverse relation of GA to circulating Tie2 and Scl/Tal1 mRNA levels. These findings indicating possible increased angioblastic activity in the younger preterm correlates with the well-established clinical observation that this specific population demonstrates higher frequency of cutaneous vascular lesions compared to older preterms and mature babies.

Brain echogenicity was associated with higher circulating *Tie2 *levels in our neonatal population. Increased echogenicity has been shown to represent an intracerebral lesion of an ischemic nature [28] whereas sustained echogenicity may have neurodevelopment consequences [30]. Our finding may indicate that C-RNA levels, mainly *Tie2*, are involved in the evolution of such ischemic lesions. This is in agreement with previous studies showing elevated levels of angiogenic markers in patients with ischemic stroke [13]. Interestingly when grouped together, infants with vascular-related morbidities (e.g., bronchopulmonary dysplasia, retinopathy of prematurity, intraventricular hemorrhage and skin hemangioma) have shown a trend towards higher *Tie2 *RNA levels but due to the small sample size we could not achieve statistical differences.

Tie2 is a transmembrane tyrosine kinase protein expressed principally on vascular endothelium which plays a critical role in vascular development [31,32]. Disruption of Tie2 function in mice resulted in embryonic lethality associated with defects in embryonic vasculature, suggesting a role for Tie2 in blood vessel maturation [33,34]. Moreover, an activating mutation in the intracellular kinase domain of the human Tie2 gene has been implicated in one form of inherited cutaneo-mucosal venous malformations, further demonstrating the critical function of Tie2 in vascular development. Over expression of Ang1, the Tie2 ligand, in mice can increase vascularization in the skin, whereas delivery of soluble Tie2 can inhibit tumor angiogenesis [35,34]. Both findings demonstrate that the activation of the angiopoietin/Tie2 signaling pathway can lead to excessive angiogenesis. Interestingly, up-regulation of Tie2 mRNA and protein levels, but not of members of the VEGF/VEGF receptor family, has been observed in most hemangioma-derived endothelial cells [34]. This and other studies [35] are consistent with our finding of the exclusive changes in Tie2 levels but not in the other markers. Thus, we hypothesize that an increased C-RNA level, reflected by elevated Tie2, contribute and/or reflects the formation of excessive and disorganized vasculature associated with brain echogenicity lesion and possibly with other progressive and proliferating vascular lesions found in BPD, ROP and PVL. Further experimental studies at the tissue levels in murine models are required to substantiate this hypothesis.

At present, it is not clear whether the elevated circulating Tie2 transcripts originate from BM-derived endothelial progenitor cells or, alternatively, if they represent immature tissue angioblasts or more mature endothelial cells that are shed from the vessel wall into the circulation during vasculogenesis. There is emerging evidence that cells expressing the combination of CD31, CD45, and Tie-2, and not CD133/Flk1, comprise a discrete population of BM-endothelial progenitor cells [36]. Clearly, as the study only presents RT-PCR data, the cellular origin of the detected circulating Tie2 mRNA needs to be further investigated and there is a possibility that multiple cell types contribute to elevated Tie2 mRNA levels.

## Conclusion

In conclusion, the findings of the current study suggest that angiogenic potential might be demonstrated in relatively higher circulating RNA markers in young preterm infants and might correlate with the appearance of brain pathology. Due to the limitation of the restricted study design in this small preterm population, further prospective follow up of larger cohorts should define the potential associations between these laboratory parameters studied, specifically Tie2 levels, as well as validate the potential to serve as a surrogate marker for preterm complications and neurodevelopment outcome

## Abbreviations

**EPC**: endothelial progenitor cell; **ELBW**: extremely low birthweight infants; **BPD**: bronchopulmonary dysplasia; **ROP**: retinopathy of prematurity; **GM-IVH**: germinal matrix, intraventricular hemorrhage; **PVL**: periventricular leukomalacia; **RT-PCR**: reverse transcription-polymerase chain reaction; **GA**: gestational age; **BW**: birthweight; **RDS**: respiratory distress syndrome; **PDA**: patent ductus arteriosus; **US**: ultrasound; **C-RNA**: circulating RNA levels

## Competing interests

The authors declare that they have no competing interests.

## Authors' contributions

TS conceived of the study, and participated in its design and coordination; SM carried out the molecular genetic studies, IP participated in its design and coordination, IS performed the statistical analysis JK participated in its design and coordination, BD conceived of the study, participated in its design and drafted the manuscript. All authors read and approved the final manuscript.

## Pre-publication history

The pre-publication history for this paper can be accessed here:


